# TDP43 and huntingtin Exon-1 undergo a conformationally specific interaction that strongly alters the fibril formation of both proteins

**DOI:** 10.1016/j.jbc.2024.107660

**Published:** 2024-08-13

**Authors:** Gincy George, Anakha Ajayan, Jobin Varkey, Nitin K. Pandey, Jeannie Chen, Ralf Langen

**Affiliations:** Physiology and Neuroscience, Zilkha Neurogenetic Institute, Keck School of Medicine, University of Southern California, Los Angeles, California, USA

**Keywords:** Huntington’s disease, ALS, huntingtin exon-1, TDP43, protein aggregation, coaggregation, electron paramagnetic resonance, condensates

## Abstract

Protein aggregation is a common feature of many neurodegenerative diseases. In Huntington’s disease, mutant huntingtin is the primary aggregating protein, but the aggregation of other proteins, such as TDP43, is likely to further contribute to toxicity. Moreover, mutant huntingtin is also a risk factor for TDP pathology in ALS. Despite this co-pathology of huntingtin and TDP43, it remains unknown whether these amyloidogenic proteins directly interact with each other. Using a combination of biophysical methods, we show that the aggregation-prone regions of both proteins, huntingtin exon-1 (Httex1) and the TDP43 low complexity domain (TDP43-LCD), interact in a conformationally specific manner. This interaction significantly slows Httex1 aggregation, while it accelerates TDP43-LCD aggregation. A key intermediate responsible for both effects is a complex formed by liquid TDP43-LCD condensates and Httex1 fibrils. This complex shields seeding competent surfaces of Httex1 fibrils from Httex1 monomers, which are excluded from the condensates. In contrast, TDP43-LCD condensates undergo an accelerated liquid-to-solid transition upon exposure to Httex1 fibrils. Cellular studies show co-aggregation of untagged Httex1 with TDP43. This interaction causes mislocalization of TDP43, which has been linked to TDP43 toxicity. The protection from Httex1 aggregation *in lieu* of TDP43-LCD aggregation is interesting, as it mirrors what has been found in disease models, namely that TDP43 can protect from huntingtin toxicity, while mutant huntingtin can promote TDP43 pathology. These results suggest that direct protein interaction could, at least in part, be responsible for the linked pathologies of both proteins.

Huntington’s disease (HD) is a neurodegenerative disorder caused by abnormal CAG expansions in the exon1 (Httex1) of huntingtin, giving rise to a protein with a stretch of 36 or more consecutive Gln (polyQ) residues (1, 2, 3, 4). Mutant Httex1 is generated by aberrant splicing of huntingtin with abnormal CAG expansions, and it is sufficient to induce disease in HD animal models (5). In disease, it is found to misfold into various oligomeric species and amyloid-like fibrils, which deposit inside affected neurons. Some of these misfolded species are thought to exert cytotoxicity, potentially by disrupting cellular processes, including transcription (6, 7, 8), protein homeostasis (9, 10, 11), and RNA metabolism (12). Mutant huntingtin aggregates can sequester and interfere with the function of proteins such as RNA-binding proteins (13), and transcription factors (14, 15, 16). Among the proteins found in Httex1 aggregates is the Tar-DNA binding protein (TDP43). TDP43 is a nuclear RNA-binding protein involved in amyotrophic lateral sclerosis (ALS), limbic-predominant age-related TDP-43 encephalopathy (LATE), and frontotemporal lobar degeneration (FTLD) (17, 18, 19, 20). In these diseases, TDP43 and its C-terminal fragments containing the low complexity domain (LCD) mislocalize into cytoplasmic inclusions (21, 22). PolyQ expansions are amyloidogenic and they are increasingly recognized as a risk factor for ALS (23, 24, 25, 26). Moreover, some HD patients show ALS-like clinical symptoms (23, 27, 28, 29, 30, 31). Interestingly, TDP43 also mislocalizes nuclear and cytoplasmic huntingtin-containing inclusions in human HD and HD R6/2 transgenic mouse brains (32, 33, 34, 35). However, it remains unclear how the TDP43-huntingtin co-localization occurs and whether it is driven by a direct interaction between the two aggregation-prone proteins.

TDP43 and Httex1 share many similarities in terms of their misfolding properties. Httex1 monomers with expanded polyQ can form oligomers, protofibrils, and fibrils (36, 37, 38, 39, 40, 41, 42, 43, 44). The early aggregation intermediates, such as oligomers, protofibrils, and unbundled fibrils are believed to be more toxic than the bundled fibrils (44, 45, 46, 47, 48, 49). Like Httex1, the low-complexity C-terminal fragment of TDP43 (TDP43-LCD) can also form oligomers and protofibrils prior to the formation of fibrils (50, 51). The similarities between the two proteins only partially extend to liquid-liquid phase separation (LLPS), a process that significantly affects fibrillization (52, 53, 54, 55, 56, 57). While TDP43 readily undergoes LLPS (55, 56, 57), Httex1 requires crowding agents as well as a C-terminal fluorescent protein tag, such as GFP or RFP, for LLPS formation (54).

Here we sought to examine whether mutant Httex1 and TDP43 can physically interact, which of the respective conformers are involved, and how these interactions impact the misfolding of the respective proteins. The answer to these questions could shed light on the origin of huntingtin and TDP43 colocalization, as well as the linked pathologies of these proteins. We, therefore, evaluated the interaction between different Httex1 and TDP43-LCD conformers and the resulting effects on their respective misfolding propensities through a combination of biophysical, biochemical, and cell biological techniques.

## Results

### Httex1 aggregates sequester cytoplasmic TDP43

**Figure 1:**
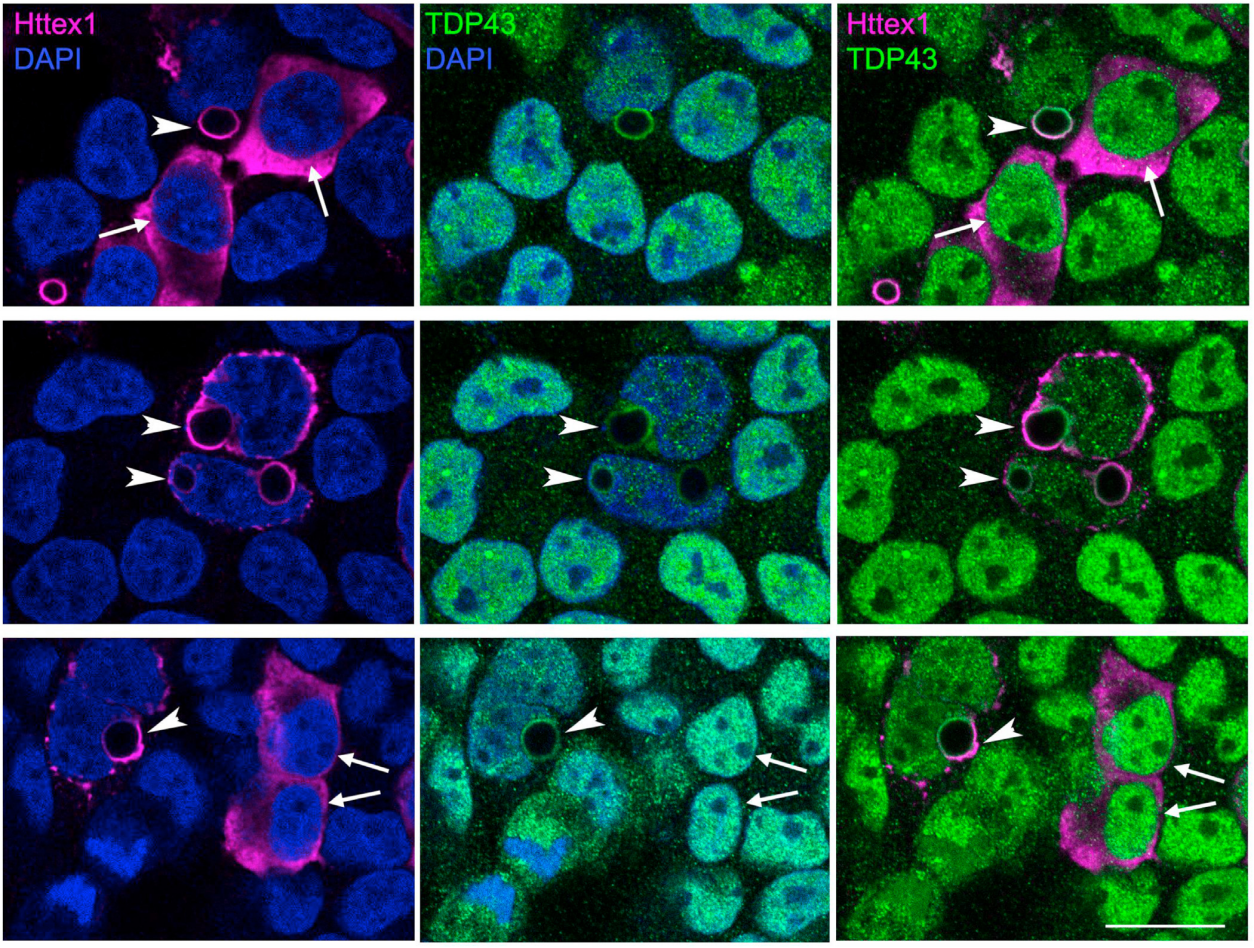
**Misfolded Httex1 causes nuclear depletion and sequestration of endogenous TDP43 to Httex1 aggregates.** Representative images of HEK293T cells 24 h after transfection with Httex1(Q72) expression construct. Httex1 is visualized by PHP1 antibody (*magenta*), endogenous TDP43 is visualized by TDP43 antibody (*green*) and cell nuclei are visualized by DAPI counterstain (*blue*). The arrows highlight cells with cytoplasmic Httex1(Q72) and normal nuclear TDP43 labeling. Arrowheads point to cells with aggregates where Httex1(Q72) and TDP43 colocalize and TDP43 signal appears depleted from the nucleus. Scale bar is 20 μm for all the panels.

Cellular interactions between Httex1 and TDP43 are often examined using fluorescently tagged proteins (58, 59, 60). A caveat of this approach is that tagging with genetically encoded fluorescent proteins may alter the biophysical characteristics and/or misfolding steps of the protein under investigation. For example, we observed that the addition of an RFP tag significantly promoted liquid-liquid phase separation followed by rapid fibrillization of Httex1-RFP whereas the untagged protein did not undergo a liquid phase during the aggregation process (54). When expressed in cells, the tagged Httex1 aggregates exhibit a different morphology and antibody reactivity as compared to the untagged Httex1 aggregates (54, 61), suggesting that they may be structurally distinct. To investigate whether untagged Httex1 recruits TDP43, we expressed Httex1(Q72) in HEK293T cells and visualized Httex1 and endogenous TDP43 using immunofluorescence microscopy (Fig. 1). Httex1(Q72) readily formed round aggregates in some transfected cells which were labeled by PHP1, an antibody that recognizes the proline-rich domain of Httex1 (62) (Fig. 1, arrowheads). As previously shown, the PHP1 fluorescent signal was restricted to the periphery of the aggregates, presumably due to limited access to the interior of the dense structures (54). Some transfected cells showed diffuse cytoplasmic labeling, likely reflecting monomeric Httex1(Q72). In cells with round aggregates, TDP43 reactivity largely co-localized with Httex1(Q72), while its normal nuclear signal was strongly reduced (Fig. 1, arrowheads). In contrast, cells with diffuse cytoplasmic Httex1(Q72) did not exhibit nuclear depletion of TDP43 and thus TDP43 did not co-localize with Httex1 (Fig. 1, arrows). These results show that misfolded Httex1 without any fluorescent protein tags recruits TDP43, thereby reducing freely available nuclear TDP43.

### Httex1 monomers do not interact with TDP43-LCD

**Figure 2:**
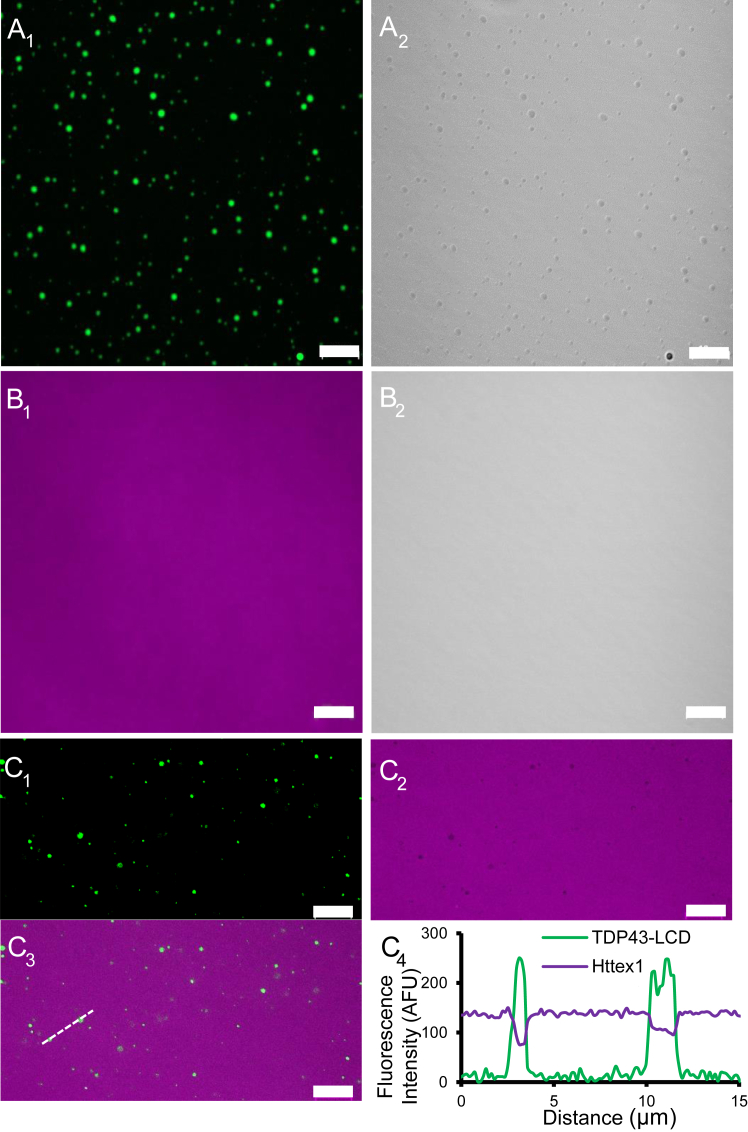
**Httex1(Q46) monomers are excluded from TDP43-LCD condensates.** Fluorescence (*A*_*1*_) and DIC (*A*_*2*_) images of 2% Alexa Fluor 488 labeled TDP43-LCD alone at 50 μM reveal rounded puncta that are typical for TDP43 condensates. Fluorescence (*B*_*1*_) and DIC imaging (*B*_*2*_) show a homogeneous distribution of 2% Alexa Fluor 594-labeled Httex1(Q46) monomer (magenta) alone, suggesting it does not undergo detectable phase separation at 10 μM. When 50 μM of Alexa Fluor 488 labeled TDP43-LCD are mixed with 10 μM of freshly prepared Alexa Fluor 594 labeled Httex1(Q46), TDP-43-LCD retains its ability to phase separate (see *green* puncta in *C*_*1*_). However, Httex1 (*magenta*) is largely excluded from TDP43-LCD condensates (*C*_*2*_), as illustrated with the merged images (*C*_*3*_). C_4_ shows a quantification of the fluorescence intensities of TDP43-LCD (*green*) and Httex1(Q46) (*magenta*) along the line shown in C_3_. Note that the Httex1(Q46) signal does not approach 0 at the location of the condensates. This would have not been expected, as Httex1(Q46)-containing bulk solution was present above and below the TDP43-LCD condensates, contributing to the signal. A more detailed quantification of the amounts of monomeric Httex1(Q46) in condensates is given in Figure 4. The scale bars are 10 μm. Images are representative of at least three independent experiments.

We used purified recombinant proteins to investigate direct physical interactions between Httex1 and TDP43. It has previously been shown that the low complexity domain of TDP43 (TDP43-LCD) is sufficient for phase separation. We therefore used TDP43-LCD, which can be readily purified from recombinant sources, for studying the Httex1-TDP43 interaction. First, we investigated the interactions of the freshly prepared proteins prior to aggregation into fibrils. To avoid label-induced artifacts, a small amount of Alexa Fluor-labeled protein (2%) was spiked into the respective unlabeled proteins. Fluorescence microscopy of TDP43-LCD sparsely labeled with Alexa Fluor 488 showed that this protein gave rise to rounded structures (Fig. 2, *A*_1_ and *A*_2_), consistent with reports that TDP43-LCD undergoes LLPS at physiological pH without the need for any crowding agents (55). In contrast, no visible phase separation was observed for freshly prepared, largely monomeric Httex1(Q46) in the absence of TDP43-LCD (Fig. 2 *B*_1_ and *B*_2_). This is also consistent with prior studies, which indicated that Httex1 phase separation requires crowding agents and C-terminal RFP or GFP tags (54). When the two proteins were mixed, TDP43-LCD retained its ability to undergo phase separation (Fig. 2*C*_1_). Interestingly, TDP43-LCD not only failed to recruit Httex1(Q46) into its condensates, but Httex1(Q46) was actively excluded from phase separated TDP43-LCD (Fig. 2 C1, C_2_, C_3_). This can be seen from the dark spots for Httex1(Q46) (Fig. 2, *C*_2,_ and *C*_3_) at locations where TDP43-LCD condensates were present (for quantification see Fig. 2*C*_4_).

### Httex1 fibrils interact with TDP43-LCD

**Figure 3:**
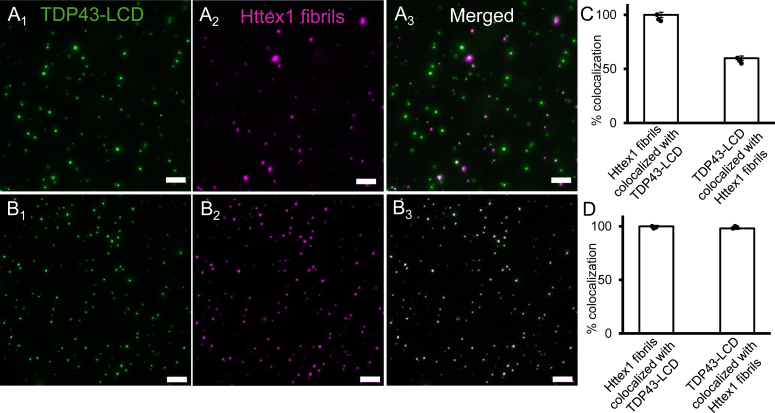
**Httex1 fibrils colocalize with TDP43-LCD condensates.** (*A*_*1-3*_) Fluorescence microscopy images of a freshly prepared mixture of 5 μM Alexa Fluor 594 labeled Httex1(Q46) fibrils (*magenta*) in the presence of 10-fold excess (50 μM) of Alexa Fluor 488 labeled TDP43-LCD condensates (*green*). Most Httex1-Q46 fibrils colocalize with TDP43-LCD (*A*_*2-3*_). The colocalization is further quantified in (*C*) using a bar graph showing the percentage of colocalization between Httex1 fibrils and TDP43-LCD and *vice versa*. While nearly 100% of the Httex1 fibrils colocalized with TDP43-LCD condensates, about 60% of the TDP43-LCD condensates contained fibrils. (*B*) The experiments in (*A*) were repeated with 2-fold excess of TDP43-LCD by mixing 10 μM Alexa Fluor 594 labeled Httex1(Q46) fibrils (B2, *magenta*) with 20 μM Alexa Fluor 488 labeled TDP43-LCD (B1, *green*). The colocalization can be seen in the merged image (*B*_*3*_). The quantification in (*D*) reveals nearly 100% colocalization of Httex1 fibrils and TDP43-LCD condensates. Scale bars are 10 μm. Images in 3A and 3B are representative of at least three independent experiments, all of which were used for the bar graphs and error bars in 3C and 3D.

Having shown that the Httex1(Q46) monomers are largely excluded from TDP43-LCD condensates, we next examined whether Httex1(Q46) fibrils might behave differently. Toward this end, we incubated 50 μM freshly prepared TDP43-LCD with 5 μM Httex1(Q46) fibrils and tested for colocalization using fluorescence microscopy. Under these conditions (ten-fold excess of TDP43-LCD), nearly all Httex1(Q46) fibrils co-localized with TDP43-LCD condensates, but many of the TDP43-LCD condensates did not contain detectable Httex1 fibrils (Fig. 3, *A*_1-3_ and C). When the Httex1(Q46)/TDP43-LCD ratio was raised to 1:2, almost complete colocalization was observed (Fig. 3 *B*_1-3_ and D). Thus, in contrast to Httex1(Q46) monomers, fibrils bind to TDP43-LCD condensates in a dose-dependent manner.

### TDP43-LCD condensates pull down Httex1 fibrils, but not Httex1 monomers

**Figure 4:**
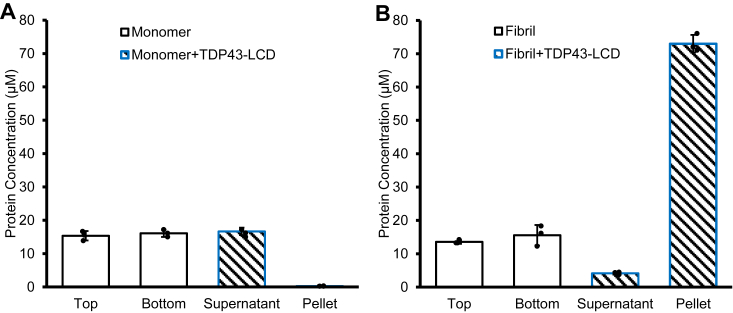
**Httex1(Q46) fibrils, but not monomers co-precipitate with TDP43-LCD condensates.** The co-precipitation of 35R1 spin-labeled Httex1 (*A*) monomers and *(B*) fibrils with TDP43-LCD condensates was quantified by EPR spectroscopy. (*A*) In the absence of TDP43-LCD condensates, no pellet was observed and an equal concentration of Httex1(Q46)-35R1 monomer was in the *top* and *bottom* regions of the centrifuged tubes (solid bars). In the presence of TDP43-LCD condensates, Httex1 monomers were almost exclusively in the supernatant and not in the TDP43-LCD condensate-containing pellet (striped bars). Panel (*B*) shows the results from a pull-down of Httex1(Q46)-35R1 fibrils with TDP43-LCD condensates (striped bars). Fibrils were strongly enriched in the pellet fraction. The control experiment in the absence of TDP43-LCD condensates (solid bars) show that the concentration of Httex1(Q46)-35R1 fibrils in the top volume and bottom volume are nearly identical. The concentration of TDP43-LCD was 50 μM and the starting concentration of Httex1(Q46) fibrils or monomers was 20 μM. Data points in the bar graph are from three independent experiments.

To further validate the imaging results, we tested whether Httex1(Q46) monomers or fibrils can co-pellet with TDP43-LCD condensates. This readout is based on the observation that TDP43-LCD condensates readily form a transparent pellet at 123,000*g*. The amounts of Httex1(Q46) monomer and fibril in the pellet and supernatant were quantified using EPR by virtue of a spin label at position 35 in the polyQ, referred to as 35R1. As a control, we tested whether Httex1(Q46)-35R1 monomers pelleted in the absence of TDP43-LCD. As expected for the soluble, monomeric protein, nearly identical concentrations were obtained for freshly prepared Httex1(Q46)-35R1 in the top half and bottom half volume of the test tubes after centrifugation (Fig. 4*A*). When Httex1(Q46)-35R1 monomer was centrifuged in the presence of TDP43-LCD condensates, a relatively low concentration of Httex1(Q46) was detected in the pellet (<0.3 μM), while that of the supernatant was much higher (16 μM) (Fig. 4*A*). Thus, Httex1(Q46) monomer was largely excluded from the pellet volume, which was almost entirely taken up by TDP43-LCD condensates. This result is consistent with the imaging experiments, which showed that monomeric Httex1(Q46) was unable to partition into TDP43-LCD condensates in any appreciable manner.

Next, the experiments were repeated with Httex1(Q46)-35R1 fibrils that had been sonicated and unbundled (see Methods). Due to their small size, these fibrils did not pellet significantly on their own. In fact, they showed nearly identical concentrations in the top volume and bottom volume, when spun in the absence of TDP43-LCD condensates (Fig. 4*B*). However, in the presence of TDP43-LCD condensates, the vast majority of the Httex1(Q46)-35R1 fibrils were in the pellet fraction (Fig. 4*B*). These data further support the notion that Httex1(Q46) fibrils, but not monomers bind to TDP43-LCD condensates. These results are furthermore consistent with our cell data, which show that fibrillar aggregates, but not diffusive, presumably monomeric Httex1 sequester TDP43 (Fig. 1).

### TDP43-LCD inhibits Httex1(Q46) aggregation

**Figure 5:**
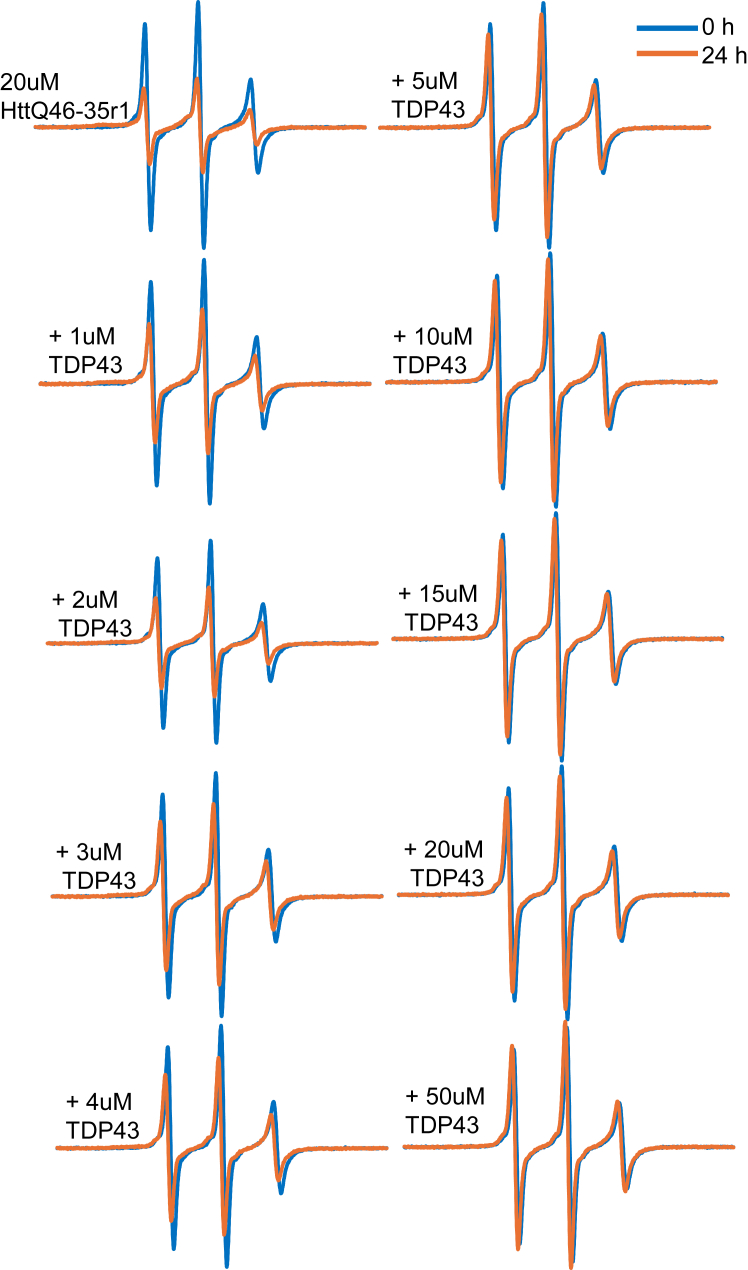
**TDP43-LCD inhibits Httex1(Q46) aggregation in sub-stoichiometric manner.** EPR spectra of 20 μM Httex1(Q46)-35R1 in the presence of the indicated concentrations of TDP43-LCD. The drop in spectral amplitude when comparing the spectra at 0 h (*blue*) and 24 h (*orange*) is a measure of Httex1(Q46)-35R1 aggregation over time, which is reduced by increasing amounts of TDP43-LCD.

To test whether the mixing of TDP43-LCD with Httex1(Q46) fibrils altered Httex1(Q46) aggregation, we employed an EPR-based aggregation readout (39). This readout leverages the characteristic spectral changes that occur as spin-labeled sites in the polyQ, such as Httex1(Q46)-35R1, change from their relatively mobile state in the monomer to an immobile state with spin-spin coupling in fibril (39). This spectral change is accompanied by a pronounced reduction in intensity, which can be conveniently monitored to follow the aggregation kinetics (39). In the absence of TDP-43, Httex1(Q46)-35R1 at 20 μM concentration underwent significant aggregation, as evidenced by the strong reduction of the EPR amplitude after 24 h (Fig. 5-top left). In contrast, the addition of TDP43-LCD slowed aggregation and reduced the concomitant amplitude loss in a dose-dependent manner (Fig. 5). While 1 μM TDP43-LCD already led to a noticeable reduction in Httex1(Q46)-35R1 aggregation, close to complete inhibition was observed in the presence of 5 μM TDP43-LCD. This is a remarkably strong inhibition, especially considering that TDP43-LCD is sub-stoichiometric relative to the 4-fold excess of Httex1(Q46).

**Figure 6:**
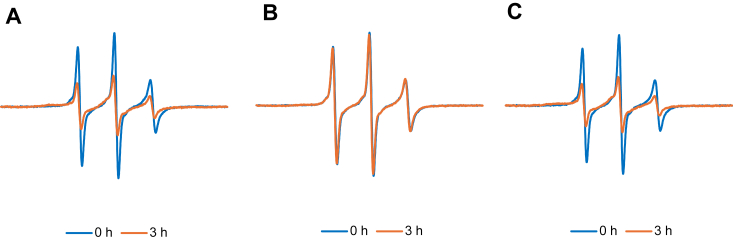
**Inhibition of seeded Httex1(Q46) aggregation by TDP43-LCD condensates, but not fibrils.** Aggregation in the presence of 5% Httex1(Q46) fibrils was monitored by EPR using the Httex1(Q46)-35R1 derivative. EPR spectra were obtained at 0 h (*blue*), and 3 h (*orange*) in (*A*) the absence or presence of (*B*) of 20 μM TDP43-LCD condensates or (*C*) 20 μM TDP43-LCD fibrils. The presence of TDP43-LCD condensates almost completely prevented seeded aggregation of Httex1(Q46)-35R1, while preformed fibrils of TDP43-LCD had no such effect. The Httex1(Q46) concentration was 20 μM.

Since TDP-43-LCD interacted with Httex1(Q46) fibrils, but not monomers, it appeared likely that TDP-43-LCD inhibited Httex1(Q46) aggregation by preventing access to seeding-competent surfaces on Httex1(Q46) fibrils. To further investigate whether TDP43-LCD interfered with seeded aggregation of Httex1(Q46), we repeated the EPR-based aggregation measurements in the presence of 5% Httex1(Q46) sonicated fibrils, which were used as seeds. In the absence of TDP43-LCD, the Httex1(Q46) aggregation was very rapid and largely complete after 3 h (Fig. 6*A*). In the presence of TDP43-LCD condensates, however, only minor spectral changes were observed (Fig. 6*B*). These data indicated that freshly prepared TDP43-LCD potently inhibited the seeding process, consistent with the ability of TDP43-LCD condensates to bind and sequester fibrils. As a control, we also tested whether pre-aggregated, fibrillar TDP43-LCD can inhibit Httex1 aggregation. However, the addition of TDP43-LCD fibrils did not reveal an inhibitory effect (Fig. 6*C*).

### Httex1 fibrils promote the aggregation of TDP43-LCD

**Figure 7:**
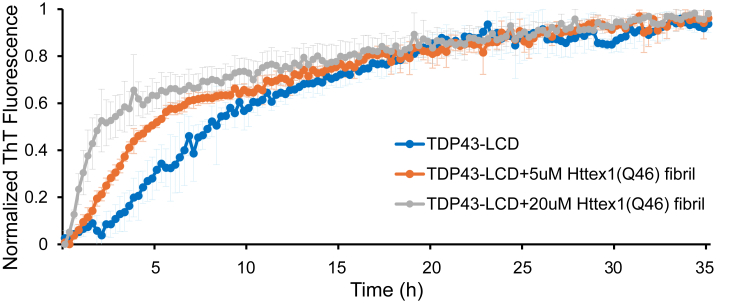
**Httex1(Q46) fibrils promote the aggregation of TDP43-LCD.** ThT fluorescence of 50 μM TDP43-LCD alone (*blue*), 50 μM TDP43-LCD in the presence of 5 μM Httex1 fibrils (*orange*), and 50 μM TDP43-LCD in the presence of 20 μM Httex1 fibrils (*grey*). ThT curves were normalized by setting their starting values to 0 and the final value at 35 h to 1. Error bars represent the standard deviation of the mean obtained from at least three independent measurements.

Having shown that the Httex1-TDP43 interaction strongly inhibits Httex1(Q46) aggregation, we next explored the reverse scenario and asked whether TDP-43 aggregation might be altered by the presence of Httex1(Q46) fibrils. Towards this end, we employed ThT fluorescence, where TDP43-LCD was incubated with increasing concentrations of Httex1(Q46) fibrils. TDP43-LCD aggregation in the absence of Httex1(Q46) fibrils gave rise to a t_1/2_ of ∼7 h (Fig. 7, blue). In the presence of increasing concentrations of Httex1(Q46) fibrils, the normalized ThT fluorescence increased, resulting in t_1/2_ values of ∼ 4 h and ∼1.5 h for 5 μM and 20 μM Httex1(Q46) fibrils, respectively (Fig. 7, orange and grey). Overall, these data indicated that TDP43-LCD aggregation was enhanced by Httex1(Q46) fibrils.

To investigate the morphological changes caused by the interaction of TDP43-LCD with Httex1(Q46) fibrils, we performed fluorescence microscopy imaging after 4 h of co-incubation. When TDP43-LCD was aged for 4 h in the absence of Httex1(Q46) fibrils, fluorescence microscopy revealed that only ∼10% of condensates became irregularly shaped (Fig. S1). In contrast, ∼60% of TDP43-LCD condensates became more irregularly shaped in the presence of Httex1(Q46) fibrils. Moreover, the formation of much larger aggregates was commonly observed in the presence of Httex1(Q46) fibrils (Fig. S1, white arrows). Interestingly, several of the irregularly shaped structures exhibited elongated protrusions (see zoomed-in insets in Fig. S1*B*). These structures were reminiscent of fibrils that have been observed to emanate from condensates in other studies on phase-separating proteins (53, 54, 55, 63, 64, 65).

To further validate the notion that Httex1 fibrils facilitate the liquid-to-solid transition of TDP43-LCD, we performed fluorescence recovery after photobleaching (FRAP). As shown in Figure S2, TDP43-LCD alone exhibited significant and rapid recovery. In contrast, the addition of Httex1(Q46) fibrils strongly reduced the amplitude and rate of recovery, consistent with a reduced liquid-like character of the TDP43-LCD condensates in the presence of Httex1(Q46).

## Discussion

Motivated by linked pathologies and colocalization of TDP43 and huntingtin in aggregates, we investigated how these proteins interact and how these interactions affect aggregation. We observed conformation-dependent interactions, which had very different effects on the aggregation of the respective proteins. While Httex1(Q46) aggregation was inhibited by TDP43-LCD, the TDP43-LCD liquid-to-solid transition was promoted by Httex1(Q46).

**Figure 8:**
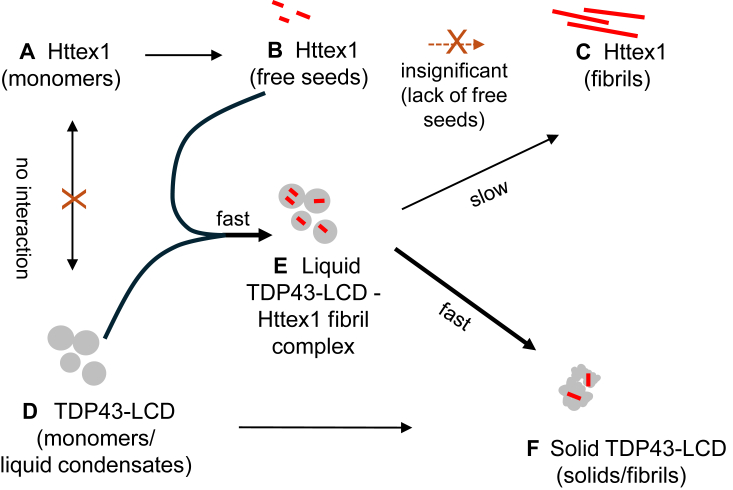
**Schematic diagram outlining the conformationally specific interactions of Httex1 and TDP-LCD.** The normal aggregation path for Httex1 (from (*A*) to (*B*) to (*C*)) and for TDP43-LCD is altered when both proteins are present together. The key intermediate that changes the aggregation pathways is the complex made up of the LLPS state of TPD43-LCD and Httex1 fibrils (*E*). This intermediate speeds up the liquid-to-solid transition (*D*–*F*) of TDP43-LCD while slowing down the aggregation of Httex1.

One might have expected that TDP43-LCD and Httex1(Q46) form hetero-coacervates that alter the respective aggregation propensities, considering prior reports showing that TDP43 and fluorescent protein-tagged Httex1 can undergo LLPS (51, 54, 55, 66, 67). However, monomeric Httex1(Q46) was largely excluded from TDP43-LCD condensates (Figs. 2 and 8) and cells with diffuse Httex1 signal showed little to no co-localization with TDP43 (Fig. 1). In contrast to Httex1(Q46) monomers, Httex1(Q46) fibrils readily interacted with TDP43-LCD condensates (Fig. 8 *A*, *B*, and *E*), and this interaction altered the aggregation of both Httex1 and TDP43-LCD. TDP43-LCD strongly inhibited the misfolding of Httex1(Q46) at concentrations that are sub-stoichiometric relative to the free Httex1(Q46) monomer concentrations. These data further illustrate that the inhibition was not mediated by stoichiometric binding to bulk monomers. Rather, our EPR data suggest that TDP43-LCD condensates interfere with the seeding process (Fig. 6*A*). In the presence of TDP43-LCD condensates, only a few Httex1 seeds are freely available to promote further aggregation (transition from B to C in Fig. 8). Instead, Httex1 fibrils or seeds are readily engulfed by TDP43-LCD condensates (B to E in Fig. 8). This interaction is likely to have two consequences. First, TDP43-LCD condensates make Httex1 seeds much less accessible to Httex1 monomers, as those are largely excluded from the condensates. This scarcity of unaggregated protein needed for fibril growth and secondary seeding is expected to slow down aggregation. Second, given the favorable interaction between Httex1 fibrils and TDP43-LCD condensates, it seems reasonable to assume that favorable interactions exist between the Httex1 fibril surfaces and TDP43-LCD condensates. This raises the possibility that seeding active surfaces on Httex1 fibrils are covered up by TDP43-LCD.

In contrast to the inhibitory effect of TDP43-LCD on Httex1(Q46) fibril formation, the opposite effect was observed for TDP43-LCD, as Httex1(Q46) fibrils accelerated the formation of thioflavinT positive structures of TDP43-LCD. The presence of Httex1(Q46) fibrils in TDP43-LCD condensates also significantly slowed the FRAP response of TDP43-LCD and caused TDP43-LCD condensates to become more irregularly shaped with rod-like, fibrillar protrusions visible in fluorescence microscopy. Together these data indicate that Httex1 fibrils promote a liquid-to-solid transition of TDP43 condensates. Critical for this liquid-to-solid transition appears to be the complex of TDP43-LCD condensates with Httex1 fibrils (Fig. 8*E*), which reduced the aggregation propensity of Httex1 (transition from E to C in Fig. 8), but which has the opposite effect on TDP43-LCD (Fig. 8, transition from E to F). At this point, it is unclear exactly how Httex1 fibrils promote the liquid-to-solid transition in TDP43 condensates. However, it is interesting to note that TDP43-LCD has regions that are Gln and Asn rich. In fact, polyQ binders used to detect Httex1, such as QBP1, also bind to TDP43 aggregates and alter TDP43 aggregation (68). Thus, the surface of TDP43 fibrils might have local regions that are structurally related to Httex1 fibrils. It may also be possible that TDP43 similarly interacts with other aggregate-forming polyQ-containing proteins, such as ataxins implicated in spinocerebellar ataxia which exhibit shared pathological features with TDP43 (24, 26, 69, 70). Whether the Gln and Asn-rich regions of TDP43 are responsible for the observed effects, and whether the present findings might also apply to other polyQ proteins still needs to be determined.

From a disease perspective, the reduction in Httex1 aggregation *in lieu* of an increased TDP43 aggregation is interesting, as it is consistent with the ability of TDP43 to reduce toxicity in HD models (58, 71). Moreover, the Httex1-dependent increase in TDP-43 aggregation might contribute to increased TDP43 toxicity. One additional aspect that is likely to contribute to TDP43 toxicity is its mislocalization from the nucleus to Httex1 aggregates (32, 33, 34, 35). Prior studies have indicated that passive diffusion plays an important role in the localization and nuclear depletion of TDP43 (72). Thus, it is likely that the affinity of TDP43 for Httex1 fibrils contributes to the TDP43 mislocalization seen *in vivo* and in our cellular studies using untagged Httex1. This mislocalization is generally thought to be a pathogenic event that contributes to TDP43 pathology (73). In addition, TDP43 mislocalization may also directly impact huntingtin toxicity. That is, a recent study used an HD mouse model and found that a reduction in nuclear TDP43 function increased polyQ expansions (74), a process that is thought to significantly contribute to huntingtin pathogenesis. Future work will be required to address these mechanisms in more detail.

In addition to the role of direct protein–protein interactions studied here, RNA interactions can further modulate TDP43 localization, LLPS, as well as aggregation into solids (75, 76). While RNA interactions typically promote TDP-43 nuclear localization (75) and liquid properties (77), some RNA interactions appear to play important roles in pathogenesis. A recent study showed that TDP43 undergoes aberrant interactions with N^1^-methyladenosine (m1A) in CAG repeat RNA, leading to mislocalization and the formation of gel-like aggregates (76). Moreover, TDP43-RNA interactions can further impact interactions with some amyloidogenic proteins (78, 79, 80, 81, 82). Future studies will have to show the extent to which various TDP43-RNA contacts might be able to further modulate the Httex1 interactions described here, especially in the context of full-length TDP43.

In summary, our study provides insights into how TDP43 and Httex1 interact and how this interaction affects the aggregation of both proteins. In addition to the potential impacts of these findings on the linked pathologies of the two proteins, the potent Httex1 aggregation inhibition observed here might point to potential new means for inhibiting Httex1 aggregation *in vivo*. Understanding the precise molecular mechanism by which TDP43 inhibits Httex1 aggregation could facilitate the development of new inhibitors that prevent aggregation of huntingtin and perhaps other polyQ proteins.

## Experimental procedures

### Expression, purification, and labeling of Httex1(Q46)

Httex1(Q46) and its single Cys mutants were expressed using the pET32a vector as described (44). Briefly, the expression plasmids encoding a Httex1(Q46)-thioredoxin fusion protein (Trx-Httex1(Q46)) were transformed into BL21(DE3) competent cells and grown overnight at 37 ˚C, followed by 50-fold dilution into LB media. Induction was done by adding 1 mM IPTG followed by incubation at 16 °C for 48 h. Cells were harvested by centrifugation at 2300*g* for 30 min. Bacteria were lysed in 20 mM Tris pH 7.4 containing 20 mM imidazole, 300 mM NaCl, and 1% Triton X-100 (Sigma) using a tip sonicator. For EPR and fluorescence experiments, cys mutants were used for covalent linkage of the respective reporter groups. For these cys mutants, 1 mM DTT was added to the lysis buffer. Lysates were separated from cell debris by centrifugation at 43,000*g* for 30 min at 4 ˚C and then incubated with NiHis60 (Takara Bio) for 30 min to bind the His-tag containing Trx-Httex1(Q46) protein. The resin was washed with 4 to 5-column volumes of wash buffer (20 mM Tris pH 7.4, 300 mM NaCl, 50 mM imidazole) for cysteine-free Httex1. For cysteine mutants Httex1(Q46)-35C (in polyQ) and Httex1(Q46)-111C (in PRD), the wash buffer contained an additional 1 mM DTT. Further, excess DTT was washed away prior to the elution using 20 mM Tris pH 7.4, 300 mM NaCl, 300 mM imidazole, 4M urea from NiHis60 beads. The same elution buffer was also used for the unlabeled Httex1(Q46). The cys mutants were labeled immediately after elution using a 5-fold molar excess of (1-oxyl-2,2,5,5-tetramethyl-Δ3-pyrroline-3-methyl) methanethiosulfonate (MTSL;Toronto Research Chemicals) to give rise to side chain R1 or Alexa-Fluor 594 (Thermo Fisher Scientific). Excess MTSL spin label or Alexa Fluor was removed using HiTrap Q XL anion-exchange column (Cytiva Life Sciences). The thioredoxin fusion tag was then cleaved by digesting the fusion protein with enterokinase (EKMax) (Thermo Fisher Scientific) for 30˚min at 32 °C. Subsequent purification was performed using the reverse-phase Phenomenex C4 column as described (39). Httex1 fractions were collected and lyophilized until further use. Httex1(Q46) was filtered using a 100 kD Amicon filter before each experiment.

### Expression and purification of TDP43-LCD

The TDP43 low complexity domain (LCD) (aa. 267–414) wild-type constructs cloned in a pRSET- B vector was a gift from Dr Witold Surewicz (55). The construct encoded the LCD protein with an N-terminal histidine tag which can be cleaved off using thrombin. The plasmid was transformed into BL21(DE3) competent cells and starter cultures were grown at 37 ˚C for 16 h followed by 50-fold dilution into LB media. For induction 1 mM IPTG was added, followed by incubation at 16 °C for 48 h. Cells were harvested by centrifugation at 2300*g* for 30 min. Bacterial cells were lysed in 8 M urea, 20 mM Tris pH 7.4, 25 mM imidazole, 300 mM NaCl containing 1% Triton ×-100 (Sigma) using a tip sonicator. For cys mutants of TDP43-LCD, 1 mM DTT was added to the lysis buffer. Lysates were separated from cell debris by centrifugation at 43,000*g* for 30 min at 4 ˚C and then incubated with NiHis60 (Takara Bio) for 30 min to bind the TDP43-LCD protein which contained an N-terminal His tag. After 30 min, the resin was washed with 4 to 5 column volumes of wash buffer (20 mM Tris pH 7.4, 300 mM NaCl, 50 mM imidazole) for TDP43-LCD. For Alexa Fluor 488 (Thermo Fisher Scientific) labeling, a cysteine was introduced at residue 311. For this cys mutant, the wash buffer contained an additional 1 mM DTT. An additional washing step using DTT-free wash buffer was added to remove excess DTT prior to the elution of the protein using 20 mM Tris pH 7.4, 300 mM NaCl, 300 mM imidazole, 8 M urea from NiHis60 beads. The cys mutant was labeled immediately with either MTSL or Alexa Fluor 488 as described for Httex1(Q46). The final purification step involved a C4 reverse column as in the case of Httex1. The TDP43-LCD-containing fraction was collected and lyophilized. For unlabeled TDP43-LCD, absorption at 280 nm was used for concentration determination using an extinction coefficient of 17,990 M^-1^ cm^-1^.

### Preparation of Httex1(Q46) fibrils

Httex1 fibrils were grown in 20 mM Tris, 150 mM NaCl pH 7.4 from a solution of 20 μM Httex1 monomers that were incubated without agitation at 4 ˚C. A previously described protocol was used to disaggregate the fibrils (39). After 20 h of incubation, fibrils were harvested at 123,000*g* for 60 min and later redissolved in a 1:4000 mixture of TFA and H_2_O. Fibrils were further disaggregated using tip sonication and concentrations were determined by CD.

### Fluorescence microscopy of recombinantly generated proteins and generation of TDP43-LCD condensates

Lyophilized TDP43-LCD was taken up in Milli-Q water and diluted into the buffer to reach a final buffer concentration of 50 mM Tris-HCl, 150 mM NaCl pH 7.4. This methodology has previously been described to be sufficient for the formation of TDP43-LCD condensates (55). Lyophilized Httex1(Q46) was taken up in 0.5% TFA/methanol for disaggregation followed by evaporation of the TFA/methanol mixture. The film was then dissolved in 50 mM Tris-HCl, and 150 mM NaCl pH 7.4. Samples were imaged using a Zeiss LSM 800 upright confocal microscope at 63x. For TDP43-LCD droplets, we mixed 2% of Alexa Fluor 488 labeled TDP43 (labeled at position 311) with unlabeled TDP43-LCD. Similarly, for Httex1 monomer, we mixed 2% of Alexa Fluor 594 labeled Httex1 (labeled at position 111) with an unlabeled Httex1 monomer. Fiji was used to quantify the fluorescence intensities in Figure 1*C*_4_. The intensity profiles were generated using the Line tool option and analyzed with Plot Profile (Fig. 1*C*_4_). The quantification in Fig. S1 was done using Fiji software. A total of 370 structures of TDP43-LCD condensates and 403 structures of TDP43-LCD in the presence of Httex1 fibrils were analyzed. The roundness of condensates was measured using a circularity value ≥ 0.9, while condensates with a value below 0.9 were considered irregular.

### Co-sedimentation of Httex1(Q46) with TDP43-LCD condensates

TDP43-LCD condensates were mixed with either Httex1 monomers (pre-filtered using 100 kD Amicon filter) or Httex1 fibrils (sonicated and unbundled). After mixing, TDP43-LCD condensates were pelleted by centrifugation at 123,000*g* at 4 ˚C. We used spin-labeled Httex1 at position 35 to measure the amount of Httex1(Q46) that co-pelleted with TDP43-LCD droplets using EPR. The control was done by measuring the concentration of the spin-labeled Httex1(Q46)-35R1 protein in the top 50% volume and bottom 50% volume of the Httex1(Q46) monomer and Httex1(Q46) sonicated fibril. Concentration determination was performed using EPR (see below).

### EPR spectroscopy

Samples were loaded in borosilicate capillaries with a 0.6 mm inner diameter. The CW-EPR spectra of freshly prepared Httex1(Q46) spin-labeled at residue 35 (Httex1(Q46)-35R1) were recorded at 20 μM on an X-band Bruker EMX EPR spectrometer fitted with a Bruker ER4119HS resonator at room temperature. An incident power of 12.7 mW along with a scan width of 100 G was used. The concentration of spin-labeled protein was measured by double integration of the EPR spectra, and the use of double integrals from concentration standards. All measurements were performed at room temperature.

### Thioflavin T(ThT) fluorescence

The aggregation kinetics of purified TDP43-LCD in the presence of Httex1(Q46) fibrils were measured by ThT fluorescence using an Eppendorf Plate Reader AF2200 equipped with appropriate filters in a Falcon 96-well (clear flat bottom) plate, over a period of 35 h at 25 °C. The excitation and emission wavelengths were 440 and 484 nm, respectively, with a slit width of 20 nm each. The concentration of TDP43-LCD was 50 μM while the concentration of Httex1(Q46) fibrils was variable as indicated in the legend. EPR was used for concentration determination of TDP43-LCD by virtue of a spin label at position 307. The fluorescence of all samples was recorded at least in triplicate. The ThT concentration was kept at 50 μM in all cases. The fluorescence intensity values were normalized to the final measurement taken at 35 h, which was set to 1.

### FRAP measurements

FRAP of TDP43-LCD was carried out using a Leica SP8 DIVE multiphoton confocal fluorescence imaging system at 63x oil-immersion objective powered by a Chameleon Discovery laser at 1050 nm (Coherent) and DMi8 inverted microscope’s external Leica 4Tune spectral hybrid detectors (emission at 519 nm for Alexa Fluor 488 fluorescence). A region of interest was selected followed by photobleaching for 4.5 s and then capturing images every 0.5 s for ∼45 s after photobleaching. The analysis of FRAP data was performed in Fiji using Stowers ImageJ plugins (https://research.stowers.org/imagejplugins/zipped_plugins.html). The intensity obtained from Fiji was normalized to the prebleach image intensity, which was set to 1.

### Cell culture and immunocytochemistry

HEK293T cells were split and seeded onto 24-well plates with poly-D-lysine coated coverslips and grown to 70% confluency. Cells were transfected with 500 ng of Httex1(Q72) plasmid DNA (Genscript) using Lipofectamine LTX with Plus Reagent transfection kit (ThermoFisher), according to the manufacturer’s protocol. Cells were allowed to express constructs for 24 h, after which the cells were fixed with 3.7% formaldehyde, permeabilized with 0.1% Triton ×, treated with 70% formic acid for 1 min, and blocked with 1% bovine serum albumin (BSA) for 1 h at room temperature. To label the Httex1 aggregates and endogenous TDP43, cells were incubated with mouse PHP1 antibody (EMD Millipore) and rabbit TDP43 polyclonal antibody (Proteintech) respectively, both at 1:500 in 0.1% BSA blocking buffer overnight at 4°C. This was followed by incubation with Alexa Fluor 488 donkey anti-rabbit and Alexa Fluor 594 donkey anti-mouse secondary antibodies (Invitrogen) at 1:200 in the same block for 1 h at room temperature. Coverslips with cells were mounted onto glass micro slides with Vectashield antifade mounting medium with DAPI (Vector Laboratories) and imaged with a Zeiss LSM 800 confocal microscope.

## Data availability

All data are contained within the manuscript.

## Supporting information

This article has supporting information.

## Conflict of interests

The authors declare that they have no conflict of interest with the contents of this article.
