# Supplementary material for: TDP43 and huntingtin Exon-1 undergo a conformationally specific interaction that strongly alters the fibril formation of both proteins
Source: J Biol Chem. 2024 Aug 13;300(9):107660. doi: 10.1016/j.jbc.2024.107660 (PMC11408864; doi:10.1016/j.jbc.2024.107660)
Supplement: Supplementary Figures [file mmc1.pdf]

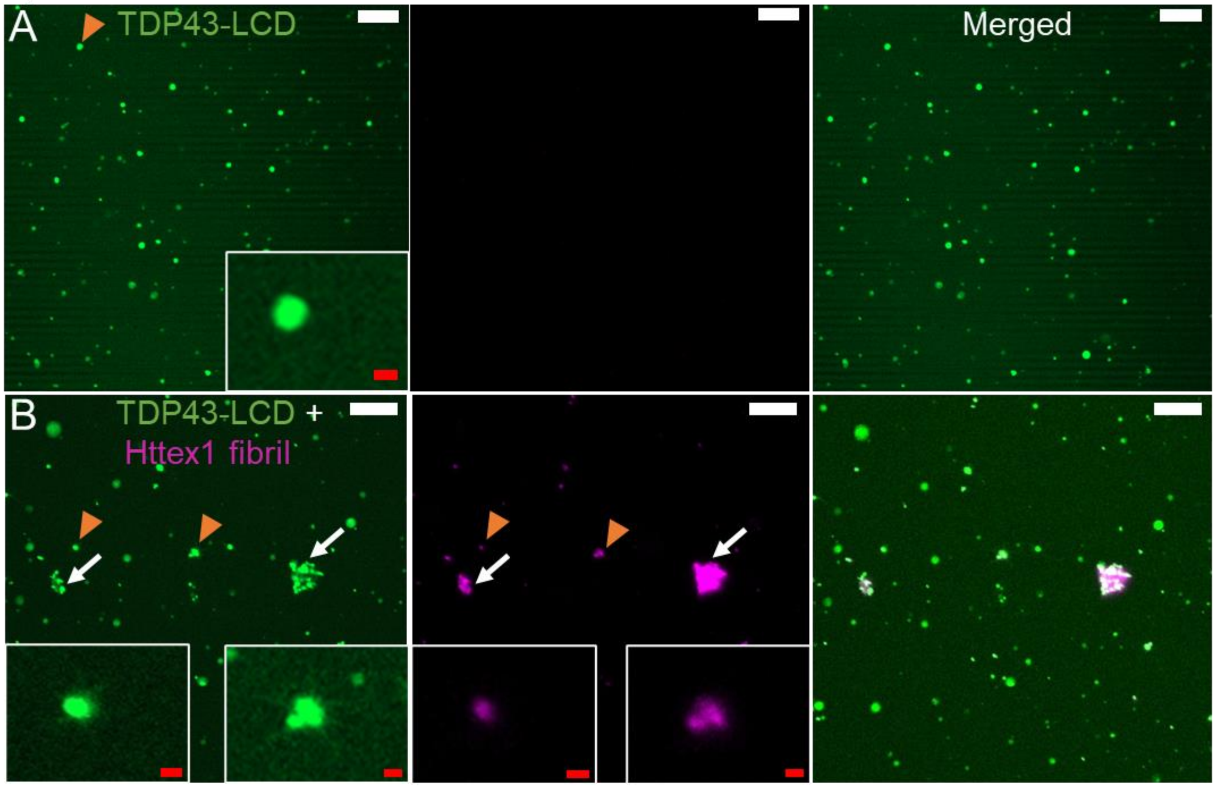

Fig S1. Httex1(Q46) fibrils promote liquid to solid transition of TDP43-LCD. Fluorescence microscopy images are shown after 4 h of incubation for (A) Alexa Fluor 488 labeled TDP43-LCD alone (green) and (B) TDP43-LCD in the presence of Alexa Fluor 594 labeled Httex1(Q46) fibrils (magenta). The TDP43-LCD aggregates in the presence of Httex1(Q46) fibrils are typically larger (white arrows) and more irregularly shaped. Puncta highlighted by orange arrowheads are shown magnified in the insets at the bottom of panels A and B. One can frequently see that TDP43-LCD puncta exhibit filamentous structures radiating outward, when in the presence of Httex1(Q46) fibrils. Protein concentrations were 5 μM for Httex1-Q46 fibril and 50 μM for TDP43-LCD. Quantification of irregular shapes was performed using Fiji (see Methods). White scale bars are 10 μm and red scale bars in the inset are 1 μm.

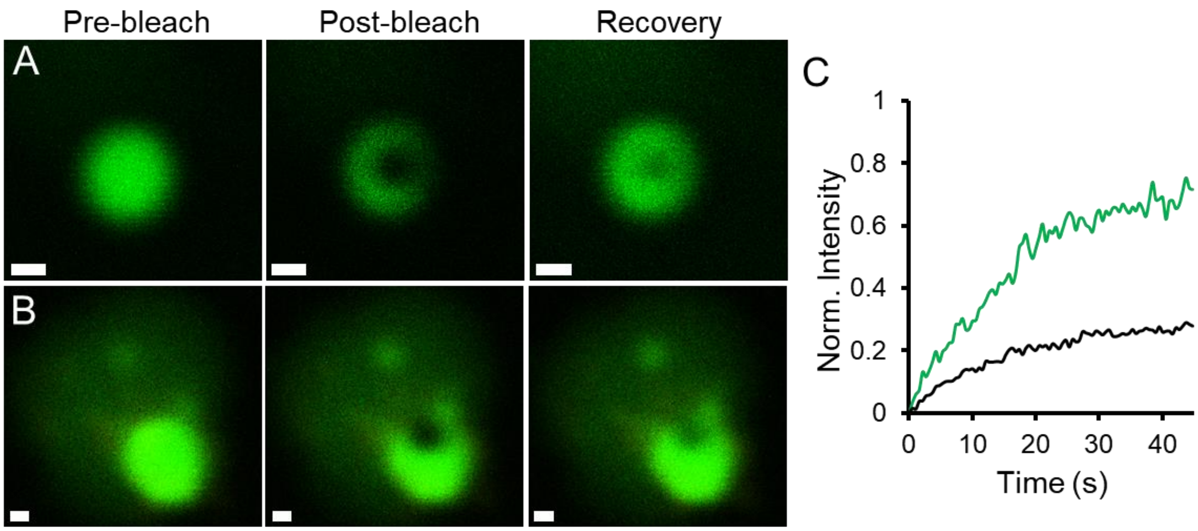

Fig. S2. Fluorescence recovery after photobleaching indicates loss of liquid-like properties of TDP43-LCD in the presence of Httex1 fibrils. FRAP experiments were performed with Alexa Fluor488 labeled TDP43-LCD incubated for 4 h alone or in the presence of Httex1 fibrils. Representative images are shown pre-bleach, post-bleach (immediately after bleach) and after ~45 seconds of recovery. Experiments were performed in the absence (A) and presence (B) of Httex1(Q46) fibrils. C) Quantification of the FRAP signals for TDP43-LCD alone (green) and TDP43-LCD in the presence of Httex1 fibrils (black). Protein concentrations were 5 µM for Httex1(Q46) fibrils and 50 µM for TDP43-LCD. Scale bars are 0.5 µm.
